# Clinical profile, reported triggers, and comorbidity pattern in children and adolescents with atopic dermatitis: a cross-sectional study from Fujian, China

**DOI:** 10.3389/fped.2026.1946416

**Published:** 2026-09-23

**Authors:** Wenping Ma, Jie Liu, Rui Li, Ziping Zhang

**Affiliations:** 1 Dermatology Department, Fuzhou First General Hospital Affiliated with Fujian Medical University, Fuzhou, Fujian, China; 2 Clinical Laboratory, Fuzhou First General Hospital Affiliated with Fujian Medical University, Fuzhou, Fujian, China; 3 Dermatology Department, The First Affiliated Hospital of Fujian Medical University, Fuzhou, Fujian, China

**Keywords:** adolescents, allergic rhinitis, atopic dermatitis, children, China, house dust mite

## Abstract

**Background:**

Detailed clinical descriptions of paediatric atopic dermatitis from southern China are scarce; available Chinese data are largely modelled burden estimates or laboratory series of allergen sensitisation.

**Methods:**

This single-centre cross-sectional study included 47 children and adolescents aged 3.6–19.0 years meeting the UK Working Party criteria at a tertiary dermatology clinic in Fuzhou, Fujian Province, China. Triggers, comorbidities and family history were extracted from routine records and are reported as recorded histories, not as verified diagnoses or as prevalence estimates. Severity was assessed by body surface area (BSA), a score the assessing dermatologist reports having assigned as a global clinical impression rather than computed from the SCORing Atopic Dermatitis (SCORAD) components, as well as the Patient-Oriented Eczema Measure (POEM) and the Children’s Dermatology Life Quality Index. The two source workbooks were audited for copying artefacts, and every value flagged by the audit was checked against the original clinic record. Proportions are reported with Wilson 95% confidence intervals.

**Results:**

Median age was 11.0 years (interquartile range 6.0–13.5 years). House dust mite was the most frequently reported trigger (27/47, 57.4%), followed by seafood or crustaceans (40.4%) and a damp environment (21.3%); most participants reported more than one trigger. No specific IgE or skin prick testing was performed, so these are reported triggers only and not sensitisations. A family history of allergic disease was recorded in 95.7% (95% CI 85.8–98.8) and allergic rhinitis in 70.2% (56.0–81.3); the rhinitis figure is partly constitutive of the enrolment criteria, and the family history figure reflects a deliberately broad definition. Recorded asthma was rare (1/44, 2.3%), a record-keeping observation rather than a prevalence estimate. Seventeen values in 10 records were residues of copying between the workbooks and were corrected against the original records. BSA and the severity score correlated within each batch (*ρ* = 0.71 and 0.58) but not when pooled (*ρ* = 0.03), whereas POEM correlated with BSA throughout; severity is therefore reported by batch.

**Conclusion:**

House dust mite was the most frequently reported trigger, a recorded history of allergic rhinitis was common, and the disease was predominantly moderate within each batch. Every figure is a recorded-history count reflecting one clinic's ascertainment and its enrolment criteria, not a population prevalence estimate. These are preliminary observations requiring confirmation in larger, prospectively defined, multi-centre cohorts.

## 1 Introduction

Atopic dermatitis (AD) is the most burdensome skin disease and affects children disproportionately. In China, Global Burden of Disease estimates place the crude prevalence highest in children under 5 years (3,456 per 100,000) and in those aged 5–9 years, with an overall age-standardised prevalence of 1,347 per 100,000 in 2021 and 15.4 million prevalent cases nationally (1, 2). Prevalence is higher in females than in males and shows a decreasing gradient from south to north and from west to east across Chinese provinces (2). Fujian lies at the high end of the first of those gradients and at the low end of the latter; therefore, its expected position cannot be inferred directly from the modelled estimates, underscoring the need for directly observed regional data.

AD is clinically and immunologically heterogeneous, and its presentation varies with age and with race or ethnicity; for example, paediatric patients show less Th1 activation than adults, and Asian patients show accentuated Th22/Th17 polarity compared with the Th2/Th22 pattern of European American patients (3). Most detailed characterisations of AD nonetheless derive from European and North American cohorts.

Chinese data on paediatric AD are dominated by modelled burden estimates (1, 2) or by large laboratory series describing allergen sensitisation (4). What is comparatively scarce is the routine-clinic view: which triggers children and their families actually report, what proportion presents with comorbid respiratory or ocular allergies, and how severity is distributed. These are the data a clinician needs to place an individual patient in context, and they are region-specific—trigger spectra in particular depend on climate and housing.

We therefore describe the clinical profile, reported triggers, comorbidity patterns, and severity distribution of children and adolescents with AD attending a tertiary dermatology clinic in Fuzhou, Fujian Province, southeastern China.

## 2 Materials and methods

This study is reported in accordance with the Strengthening the Reporting of Observational Studies in Epidemiology (STROBE) statement for cross-sectional studies; the completed checklist is provided in the Supplementary Material.

### 2.1 Study design and participants

This single-centre cross-sectional study was conducted in the Department of Dermatology, Fuzhou First General Hospital Affiliated with Fujian Medical University, a tertiary referral centre serving Fuzhou and the surrounding area of Fujian Province. Children and adolescents with AD attending the outpatient dermatology clinic were eligible. As set out below, the analysed records were assembled in two source batches in early 2025; the dates of the underlying clinical encounters were not recoverable from the source records, and exact recruitment dates can therefore not be determined.

The analysed records were drawn from two source batches, which we refer to as batch 1 and batch 2. The first batch comprised 18 patient records, of which two were removed (Section 3.1), leaving 16; the second comprised 31 records. Neither record set carries any date. The only dating evidence available was the last-modified timestamp of the two files (8 February and 26 March 2025) and the filename of the second, which referred to 25 March; we report these as the dates on which the record sets were assembled and not as the dates of the clinical encounters, which could not be recovered. The division is therefore by source batch and was not derived from the data. The source records do not identify the assessor or document a scoring procedure; upon enquiry, the assessing dermatologist reported that she assessed both record sets herself (Section 2.2). This remains a material limitation for the severity comparison (Sections 3.3 and 4.4), because the assessment was unblinded and no reliability data exist.

Inclusion criteria were a diagnosis of AD meeting the UK Working Party (Williams) criteria (5); age below 20 years; and both body surface area (BSA) and a severity score recorded at the index visit. Patients were excluded if they had received systemic corticosteroids or immunosuppressants within the preceding 3 months, or had a concomitant inflammatory skin disease, a malignancy, or an active systemic infection.

A complete screening log was not available; therefore, the number of patients assessed for eligibility before these records were compiled, and the number excluded under each criterion, cannot be reported; the 49 records thus represent those identified in the two source batches rather than a formally screened sample. We draw attention to the corticosteroid and immunosuppressant exclusion, which will have systematically removed the most severely affected patients from what is a tertiary referral clinic. Together with the fact that an outpatient cross-sectional sample generally undersamples severe disease, and considering the batch-related scoring difference described in Section 3.3, this likely explains why the maximum severity score in this series was 55.

This retrospective observational study was approved by the Ethics Committee of Fuzhou First General Hospital Affiliated with Fujian Medical University [approval no. (伦审)2026(科研)第060号; English translation: Ethics Review 2026 (Research) No. 060; approval date: 8 June 2026]. The requirement for written informed consent was waived by the Ethics Committee because of the retrospective design and the use of de-identified, routinely collected clinical data. The study was conducted in accordance with the local legislation and institutional requirements and with the Declaration of Helsinki.

### 2.2 Clinical assessment and definitions

Demographic and clinical data—including age, sex, body weight, place of residence, dietary pattern, reported triggers, comorbid allergic rhinitis, asthma and conjunctivitis, and family history of allergic disease—were retrieved from routine clinical records.

Reported triggers were recorded as clinician-documented, parent-, or patient-reported history taken at the index consultation. Neither specific IgE testing nor skin prick testing was performed, and no external test results were incorporated. These data therefore describe reported triggers and must not be interpreted as confirmed sensitisations or as sensitisation prevalence rates.

Free-text entries in the routine “allergen” field were coded into 11 categories using a written dictionary, which is reproduced in full in Supplementary Table 2. Coding was performed by a single investigator, and the dictionary was not registered in advance; it is published here so that every decision can be inspected. This field was completed for all 47 patients, and 47 is therefore the denominator for all trigger proportions. Two patients had no entries falling into any of the 11 categories: one whose record stated that no definite allergen was reported, and one whose only entry was mango, for which no category had been defined. Both were retained in the denominator, and both represent true zeros rather than missing values. Five further entries, in four patients, did not map to any category and were left uncoded: a dry environment (two patients), and unspecified hair, unspecified “food,” and the residue of one garbled entry (one patient each). The 11 categories are therefore exhaustive of what could be coded but not of what was reported—2 of the uncoded entries are food reports that appear in none of the 4 food categories—so the proportions describe the categories as defined and are lower bounds for broader groupings such as “any food.” Categories are reported without merging, but we note that three of them—damp environment, heat, and cold air—are non-specific aggravating factors rather than candidate allergens, and should be interpreted separately from the aeroallergen and food categories. Throughout this article, “reported trigger” denotes a clinician-documented, parent-, or patient-reported association only; it does not denote sensitisation, allergy, or an indication for avoidance.

A family history of allergic disease was defined as positive when the clinical notes documented any allergic condition—atopic dermatitis, allergic rhinitis, asthma, urticaria, or an unspecified allergy—in a relative of any degree; it was not verified against medical or test records, and at least four positive cases rested on a family history of urticaria alone. We therefore use “family history of allergic disease” in preference to “family history of atopy.” Allergic rhinitis, asthma, and allergic conjunctivitis were extracted from affirmative entries in the routine clinical history and were not re-diagnosed for this study; no rhinoscopy, spirometry, or bronchial challenge was performed, and they are reported as recorded histories rather than as verified diagnoses.

Disease severity was assessed at the index visit by a single dermatologist (WM), who assessed every patient in both record sets. Two clinician-assessed measures were recorded: the BSA involved, expressed as a percentage, and a severity score entered in the source documents as SCORing Atopic Dermatitis (SCORAD) (6) and classified as mild (<25), moderate (25–50), or severe (>50). Upon enquiry, the assessing dermatologist reported that this score was assigned as a global clinical impression rather than computed item by item from the SCORAD components. This account was given retrospectively; the source records neither identify the assessor nor document a scoring procedure. We therefore use the SCORAD severity bands only as descriptive cut-offs; we do not decompose the score into the terms of the formula, and we do not treat it as numerically comparable with SCORAD totals computed from the instrument; we therefore refer to it throughout as the severity score and reserve the name SCORAD for the instrument itself and for scores computed from it. Assessment was unblinded, no patient was scored by a second assessor, and neither inter- nor intra-rater reliability was measured. Patient- or carer-reported measures were the Patient-Oriented Eczema Measure (POEM) (7) and the Children's Dermatology Life Quality Index (CDLQI) (8), which the source workbook abbreviates to “DLQI” and records on a 1–4 item scale; totals are converted to the standard 0–30 scale as described in Section 3.3. Peripheral blood eosinophil data were available for only 7–10 of the 47 patients and were not analysed. A serum immunoglobulin E (IgE) result was also present in the routine records. The source records label it only as “allergen IgE,” and neither the assay platform, nor the analytical method, nor the unit is recorded; the two source batches contain different recorded reference limits, which we could not reconcile. A co-author (JL) made enquiries within the hospital's clinical laboratory, but the assay information for these records could not be located. The variable is therefore not reported in this article (Section 4.5).

### 2.3 Statistical analysis

Continuous variables are summarised as medians (interquartile ranges, IQRs) and categorical variables as counts with percentages; Wilson 95% confidence intervals are reported for proportions carrying interpretive weight. Denominators are stated for every variable because ascertainment was incomplete for several items.

Between-group comparisons were performed using the Mann–Whitney U test for continuous variables. Correlations between severity measures were evaluated using Spearman rank correlation coefficients. Differences across the three age groups were assessed using the Kruskal–Wallis test. Because severity scoring differed between the two source batches (Section 3.3), severity comparisons and correlations are reported within-batch as well as pooled, and any association not reproducible within a batch is not reported as a finding.

All analyses are descriptive, and no hypothesis was pre-specified. No correction for multiplicity was applied, and any individual *p* < 0.05 should be regarded as hypothesis-generating rather than confirmatory. At 80% power, the smallest correlation detectable in this cohort is approximately |*ρ*| = 0.40 at *n* = 47, 0.48 at *n* = 31, and 0.65 at *n* = 16; therefore, the absence of a detected association does not exclude one of moderate strength. Analyses were performed in Python 3 (pandas, SciPy, NumPy, statsmodels); the results reported here were reproduced with Python 3.9.6, pandas 2.3.3, NumPy 1.26.4, SciPy 1.13.1, and statsmodels 0.14.6. Wilson intervals were obtained with the statsmodels function proportion_confint; between-group comparisons are two-sided Mann–Whitney *U* tests; correlations are Spearman coefficients with the associated asymptotic *p*-value; and the stratified pooled correlation ranks BSA and the severity score within-batch, as percentile ranks, before pooling. The check for copying artefacts described in Section 3.1 compares the two source workbooks cell by cell at matching row positions and refers the observed number of coincidences to a permutation distribution in which the second batch's records are randomly reassigned to positions (200,000 draws). The check was applied to every analysed variable. It uses two matrices of recorded values by source-workbook row position; each holds the four recorded measures, the 11 coded trigger categories, and an arbitrary label for the free-text trigger entry, assigned so that two entries can be tested for identity without disclosing their contents. Neither matrix contains a name, an identifier, a date, an age, a sex or a place of residence, but both are individual-level records; for the reason given in the Data Availability Statement, they are not deposited publicly, and were supplied to the Editorial Office for the reviewers. The code in Supplementary Data Sheet 1 reproduces the post-correction coincidence counts and permutation tests for the four recorded measures, the eleven coded trigger categories, and the free-text trigger-entry identifiers from these matrices. So that the analysis can be repeated, the trigger coding dictionary is provided in Supplementary Table 2 and a variable-by-variable data dictionary as Supplementary Table 3. The analysis code is provided as Supplementary Data Sheet 1 and reproduces the reported results from the de-identified dataset described in the Data Availability Statement, with the following exception arising from de-identification. Exact age is released only in bands, so the age descriptive statistics in Table 1 and every comparison that uses exact age cannot be reproduced from it; the released dataset reproduces the rhinitis–age comparisons on bands instead (pooled *p* = 0.020 against 0.013 on exact ages; within-batch *p* = 0.251 and *p* = 0.059 against *p* = 0.200 and *p* = 0.042). The code produces the principal descriptive statistics and association analyses from the de-identified analysis dataset; the available scope of the provenance check is specified above. The figures that describe the data before correction—the 20 of 71 coincidences, the same-position agreement in residence, body weight and recorded rhinitis, and the counts obtained when suspected residues are set aside—are computed from the source records themselves, which are not released; of the 23 variables to which the comparison was applied, 15 can be recomputed from those two matrices and 8 cannot.

**Table 1:** Demographic, clinical, and trigger profile of the 47 children and adolescents with atopic dermatitis.

Characteristic	*n* with data	Value
Age, years, median (IQR)	47	11.0 (6.0–13.5); range 3.6–19.0
<6/6–11/≥12 years, *n* (%)	47	11 (23.4)/17 (36.2)/19 (40.4)
Male sex, *n* (%)	46	26 (56.5)
Body weight, kg, median (range)	47	35.0 (12.3–65.0)
Urban residence, *n* (%)	47	42 (89.4)
Reported triggers^a^, *n* (%)		
House dust mite	47	27 (57.4)
Seafood or crustaceans	47	19 (40.4)
Damp environment	47	10 (21.3)
Egg or milk	47	7 (14.9)
Pollen	47	7 (14.9)
Cold air	47	7 (14.9)
Animal dander	47	6 (12.8)
Heat	47	6 (12.8)
Beef or mutton	47	4 (8.5)
Wheat	47	1 (2.1)
Cockroach	47	1 (2.1)
Recorded comorbid conditions^b^		
Recorded history of allergic rhinitis, *n* (%) [95% CI]	47	33 (70.2) [56.0–81.3]
Recorded history of allergic conjunctivitis, *n* (%) [95% CI]	44	6 (13.6) [6.4–26.7]
Recorded history of asthma, *n* (%) [95% CI]	44	1 (2.3) [0.4–11.8]
≥1 respiratory or ocular comorbidity, *n* (%)	47	36 (76.6)
Family history of allergic disease, *n* (%) [95% CI]^c^	47	45 (95.7) [85.8–98.8]
Patient-reported outcomes		
POEM, median (IQR)	46	16.0 (13.2–19.0)
CDLQI, median (IQR)^d^	47	8 (6–11); range 1–27

CDLQI, Children's Dermatology Life Quality Index; CI, confidence interval (Wilson); IQR, interquartile range; POEM, Patient-Oriented Eczema Measure.

Denominators are given for every variable because ascertainment was incomplete for several items. Severity and the two patient-reported measures are reported separately by source-record batch in Table 2; see Section 3.3.

a Clinician-recorded, parent-, or patient-reported triggers documented at the index consultation. No specific IgE testing or skin prick testing was performed; these are reported triggers and must not be read as confirmed sensitisation or as sensitisation prevalence. Most patients reported more than one trigger, so proportions sum to more than 100%. The denominator is all 47 patients; the routine “allergen” field was completed for every patient. Two patients have no entry in any category, and five further entries in four patients fell outside the categories; all are listed in Supplementary Table 2.

b Rhinitis, asthma, and conjunctivitis were taken from affirmative entries in the routine clinical history and were not re-diagnosed for this study; they are recorded histories, not verified diagnoses, they are not prevalence estimates, and ascertainment differed between the two source batches (Section 4.3).

c Family history was counted as positive for any allergic condition—including urticaria—in a relative of any degree, unverified; hence “allergic disease” rather than “atopy” (Section 2.2).

d The CDLQI totals are recorded on a 1–4 item scale rather than the standard 0–3, which the assessing clinician reports having confirmed against the original questionnaire and scoring template; totals are converted to the standard 0–30 scale by subtracting 10 (Section 3.3). Eight of the 47 totals come from the electronic record rather than the workbook; the assessing clinician confirms that it stores the same unconverted totals (Section 3.3). The conversion is arithmetic and preserves every rank.

## 3 Results

### 3.1 Cohort and data quality

Forty-nine records were identified across the two source batches—18 in the first and 31 in the second. Two records, both in the first batch, were removed because their laboratory data blocks were exact duplicates of another patient's records, one of them additionally lacking all demographic entries; thus, 47 patients were analysed (16 in batch 1 and 31 in batch 2). For this revision, the two source-record sets were retrieved and every clinical variable was transcribed again from the original documents and compared with the analysis dataset value by value. For body surface area, the severity score, POEM, CDLQI, the recorded histories of rhinitis, asthma and conjunctivitis, and the family history, the two transcriptions agreed for all 47 records without exception; the pattern of missing values was also reproduced, including the three consecutive records in the second batch whose comorbidity fields were illegible. The recheck identified one coding error in the trigger field, which is corrected in this version (Sections 2.2 and 3.2). It also showed that the second workbook had been produced by copying the first and overwriting its contents, and that the overwriting was incomplete. The first row of the second batch's severity table still contained the name of the first patient from the first batch, four free-text allergen entries were identical between the two batches at the same row position—one of them a six-item entry identical token for token—and two records in the second batch were entered under inconsistent names across the four tables, one of them under three different names.

Because these are copying artefacts rather than transcription errors, a second transcription cannot detect them, and we therefore tested for them directly. Comparing the two workbooks position by position across body surface area, the severity score, POEM, and the quality-of-life score, 20 of 71 cells were identical between the two batches at the same row position, against 4.5 expected when the second batch's records are randomly reassigned to positions (permutation test, 200,000 draws, *p* < 0.000005). The patients concerned differed in name and in age, and in 9 of the 16 matched positions also in sex, so these are not duplicate records of the same patient.

Every cell identified by this comparison was referred to the assessing dermatologist, who retrieved the corresponding original electronic outpatient records and confirmed or corrected each value in writing. The same comparison was then extended to the variables that had not been part of the first enquiry, and it identified three additional variables: recorded residence agreed at 16 of 16 matched positions, body weight at 2 of 16 against 0.10 expected, and recorded allergic rhinitis at 13 of 16 against 8.9 expected. All three were referred to the clinician, and in all three the comparison proved correct. The comparison was applied to every analysed variable, although for three of the 11 coded trigger categories the second batch was constant, so the screen had no power there. In the free-text trigger entry itself, three entries remained identical token for token at the same row position after the one corrected entry, against 0.26 expected (*p* = 0.0008), and two coded trigger categories showed a nominal excess (egg or milk, agreement at 15 of 16 positions, *p* = 0.011; animal dander, 14 of 16, *p* = 0.013). We do not discount these on multiplicity grounds: recorded residence, body weight, and recorded rhinitis failed a Bonferroni threshold for the same family of 23 variables, and all three were nevertheless confirmed as residues against the original records. Six second batch entries accounted for these signals. The trigger field had not been covered by either of the first two enquiries, so all six were referred to the assessing clinician in a third enquiry, and all six were confirmed against the original electronic outpatient records exactly as entered; no trigger value was changed. The same-position agreement in this field is therefore not attributable to copying: three of the six are short entries that recur throughout the cohort, and the other three overlap only partly with the entry at the matching position. We report the test because it was the reason for the third enquiry, not as contamination that survived it. The stakes were not small. If, hypothetically, all six queried entries had instead reported seafood or crustaceans alone, house dust mite and seafood or crustaceans would each have been reported in 23 of 47 patients (48.9%).

In total, 17 values in 10 records were residues of the copying and have been corrected: 1 free-text allergen entry, which the original record documented as “no definite allergen reported”; 12 severity, POEM, or quality-of-life scores; 1 body weight; 1 place of residence; and 2 entries for allergic rhinitis, both of which the original notes recorded as present. All results in this article are computed using the corrected data. All 12 corrected severity, POEM, and quality-of-life scores moved downward (sign test *p* = 0.0005), and correcting them strengthened the between-batch difference in Table 2 from *p* = 0.0016 to *p* = 0.0007. A further undetected residue of the same kind would therefore attenuate rather than create that between-batch difference. Its effect on the within-batch correlations runs the other way: removing residues lowers them, batch 2 *ρ* falling from 0.61 before correction to 0.58 after correction; therefore, an undetected residue of this kind would inflate a within-batch correlation rather than attenuate it. We acknowledge that we defined the scope of the first enquiry too narrowly, and that it was the statistical comparison rather than our own judgement that identified the three additional fields.

**Table 2:** Severity and patient-reported measures, reported separately for the two source-record batches (Section 3.3, Figure 2).

Measure	Batch 1	Batch 2	Between-batch p or pooled estimate
BSA involved, %, median (IQR; range)	10.5 (9.8–13.2; 7–17), *n* = 16	17.0 (15.0–22.0; 11–33), *n* = 31	<0.001
Severity score (recorded as SCORAD), median (IQR; range)	44.5 (37.5–45.2; 33–55), *n* = 16	34.0 (32.0–38.0; 17–49), *n* = 31	<0.001
POEM, median (IQR; range)	15.0 (11.0–18.5; 9–22), *n* = 15	16.0 (15.0–19.0; 9–25), *n* = 31	0.20
CDLQI (0–30), median (IQR; range)	8 (5.0–10.2; 2–13), *n* = 16	8 (6.0–11.0; 1–27), *n* = 31	0.28
Mild (score <25), *n*	0	4	—
Moderate (25–50), *n*	13	27	—
Severe (>50), *n*	3	0	—
BSA–severity score, Spearman *ρ*	+0.71 (0.002)	+0.58 (<0.001)	naive pooled +0.03 (0.835)
POEM–BSA, Spearman *ρ*^a^	+0.75 (0.001)	+0.56 (0.001)	pooled +0.57 (<0.001)
Stratified BSA–severity score *ρ*^b^			+0.62; inverse-variance +0.63 (95% CI 0.41–0.78)

Both batches were assessed by the same dermatologist, who assigned the severity score as a global clinical impression rather than computing it from the SCORAD components (Section 2.2).

BSA, body surface area; CDLQI, Children's Dermatology Life Quality Index; POEM, Patient-Oriented Eczema Measure; SCORAD, SCORing Atopic Dermatitis.

For BSA, severity score, POEM and CDLQI, the final column gives the between-batch *p* value. For the correlation rows, it gives the pooled or stratified estimate as labelled; values in parentheses after individual correlation coefficients are *p* values unless identified as a confidence interval.

a POEM is patient- or carer-reported and therefore not subject to clinician rater drift. It retains its association with BSA both within each batch and across the pooled cohort, whereas the association between BSA and the clinician-assigned severity score does not survive naive pooling.

b BSA and the severity score ranked within batch (percentile ranks) before pooling; an inverse-variance-weighted combination of the two within-batch coefficients is given alongside it with its confidence interval. It is reported in preference to the naive pooled value, but it estimates only the rank association common to the two batches and removes by construction the twofold difference in their regression slopes (1.85 and 0.86 severity score points per BSA percentage point).

After correction, 8 of 71 same-position cells remained identical, against 4.2 expected (permutation *p* = 0.05). The 71-cell frame is that of the source workbooks and includes the two row positions whose batch 1 records were excluded from analysis; it is the frame in which the copying took place, and 2 of the 17 corrected values were indeed residues of one of those two excluded records. It is also the more conservative of the two frames, because after correction those two positions can contribute no coincidences at all: restricted to the 47 analysed records, the same residual is 8 of 63 against 3.7 expected (*p* = 0.026), a stronger signal than the 71-cell figure. We report the wider frame as the primary one and the narrower one beside it. Each was confirmed individually by the assessing dermatologist against the original record. The residual excess is nonetheless compatible with anywhere between zero and eight further undetected residues, so we do not describe the remaining agreements as coincidental. Setting the batch 2 value of all eight to missing leaves the within-batch correlations and the between-batch comparisons of Table 2 unchanged in direction and significance (batch 1 *ρ* = 0.71 unchanged, batch 2 *ρ* = 0.58–0.55; between-batch severity *p* = 0.0007–0.0004). We also note that, in addition to the two records excluded for having laboratory blocks identical to another patient's records, one retained pair shared 10 of 12 values in a laboratory panel that is not used in this analysis; the two patients differed in sex, age, body weight, body surface area, POEM, and the quality-of-life score, but shared the recorded severity score, place of residence, and all four recorded comorbidity and family history fields. Ascertainment was incomplete for several variables and denominators are reported throughout (Table 1).

### 3.2 Demographic, trigger, and comorbidity profile

The 47 patients had a median age of 11.0 years (IQR 6.0–13.5; range 3.6–19.0). Eleven (23.4%) were aged under 6 years, 17 (36.2%) were aged 6–11 years, and 19 (40.4%) were aged 12 years or older. Twenty-six of 46 (56.5%) were male, and 42 of 47 (89.4%) lived in an urban area (Table 1).

The routine “allergen” field was completed for all 47 patients, and 47 is the denominator throughout (Figure 1A). House dust mite was by far the most frequently reported trigger (27, 57.4%, 95% CI 43.3–70.5), followed by seafood or crustaceans (19, 40.4%), a damp environment (10, 21.3%), and pollen, cold air, and egg or milk (seven each, 14.9%). Animal dander and heat (six each, 12.8%), beef or mutton (four, 8.5%), wheat (one, 2.1%), and cockroach (one, 2.1%) were reported less often. Most patients reported more than one trigger; two patients had no entries in any category (Section 2.2), and five further entries in four patients fell outside the categories and were left uncoded. These proportions describe what was written in the records; they are not sensitisation frequencies and carry no implication for dietary or environmental avoidance.

**Figure 1:**
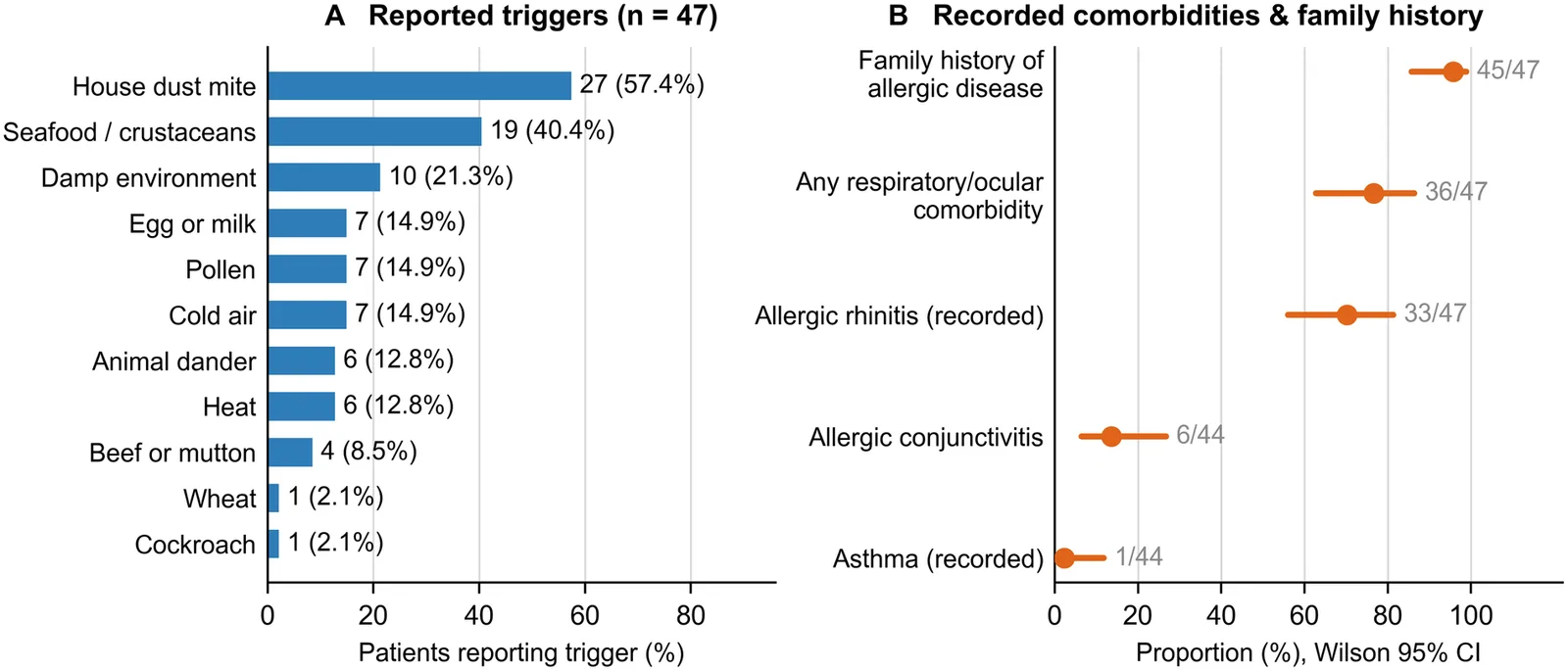
Reported triggers and recorded comorbid conditions in 47 children and adolescents with atopic dermatitis. **(A)** Triggers documented from clinician-recorded, parent- or patient-reported history (*n* = 47; the routine “allergen” field was completed for every patient, two of whom had no entry falling in any category); most patients reported more than one, so proportions sum to more than 100%. These were not confirmed by specific IgE or skin prick testing. **(B)** Recorded comorbid allergic conditions and family history of allergic disease, each shown as the proportion (dot) with its Wilson 95% confidence interval (whisker); denominators differ between items and are shown beside each estimate. The wide interval on asthma reflects a single recorded case, which is interpreted as a record-keeping observation rather than a prevalence estimate (Section 4.3). CI, confidence interval.

A recorded history of allergic rhinitis was present in 33 of 47 patients (70.2%, 95% CI 56.0–81.3) and of allergic conjunctivitis in 6 of 44 (13.6%); 36 of 47 (76.6%) had at least one recorded respiratory or ocular allergic comorbidity (Figure 1B). Asthma was recorded in only 1 of 44 patients (2.3%, 95% CI 0.4–11.8). A family history of allergic disease was recorded in 45 of 47 (95.7%, 85.8–98.8). These are recorded histories, not diagnoses re-established for this study (Section 2.2), and none of these proportions is offered as a prevalence estimate.

Patients with allergic rhinitis were older than those without (median 11.2 vs. 6.0 years, *p* = 0.013). Within batch 2, the association persisted (11.2 vs. 5.9 years, *p* = 0.042); within batch 1, it could not be reliably assessed, as only 4 of 16 patients had no rhinitis (13.0 vs. 10.0 years, *p* = 0.200).

Ascertainment of the comorbidities themselves differed between the two batches, in the same direction as the severity scoring. Allergic conjunctivitis was recorded in 0 of 16 patients in batch 1 and 6 of 28 in batch 2; recorded asthma in 0 of 16 and 1 of 28; and allergic rhinitis in 12 of 16 and 21 of 31. The pooled comorbidity proportions in Table 1 should therefore be interpreted as artefacts of record-keeping as much as clinical phenotypes, and we report the batch-specific counts alongside them.

### 3.3 Severity, and why it is reported by batch

Body surface area and the severity score correlated strongly within each source batch (*ρ* = 0.71 and 0.58) but not when the two were naively pooled (*ρ* = 0.03, *p* = 0.835; Figure 2A). Records in batch 2 had systematically larger BSA (median 17.0% vs. 10.5%, *p* < 0.001) yet a lower severity score (34.0 vs. 44.5, *p* < 0.001). Because the score was assigned as a global impression rather than computed from the SCORAD formula, we do not treat body surface area as a component of it and do not attempt to partition their association.

**Figure 2:**
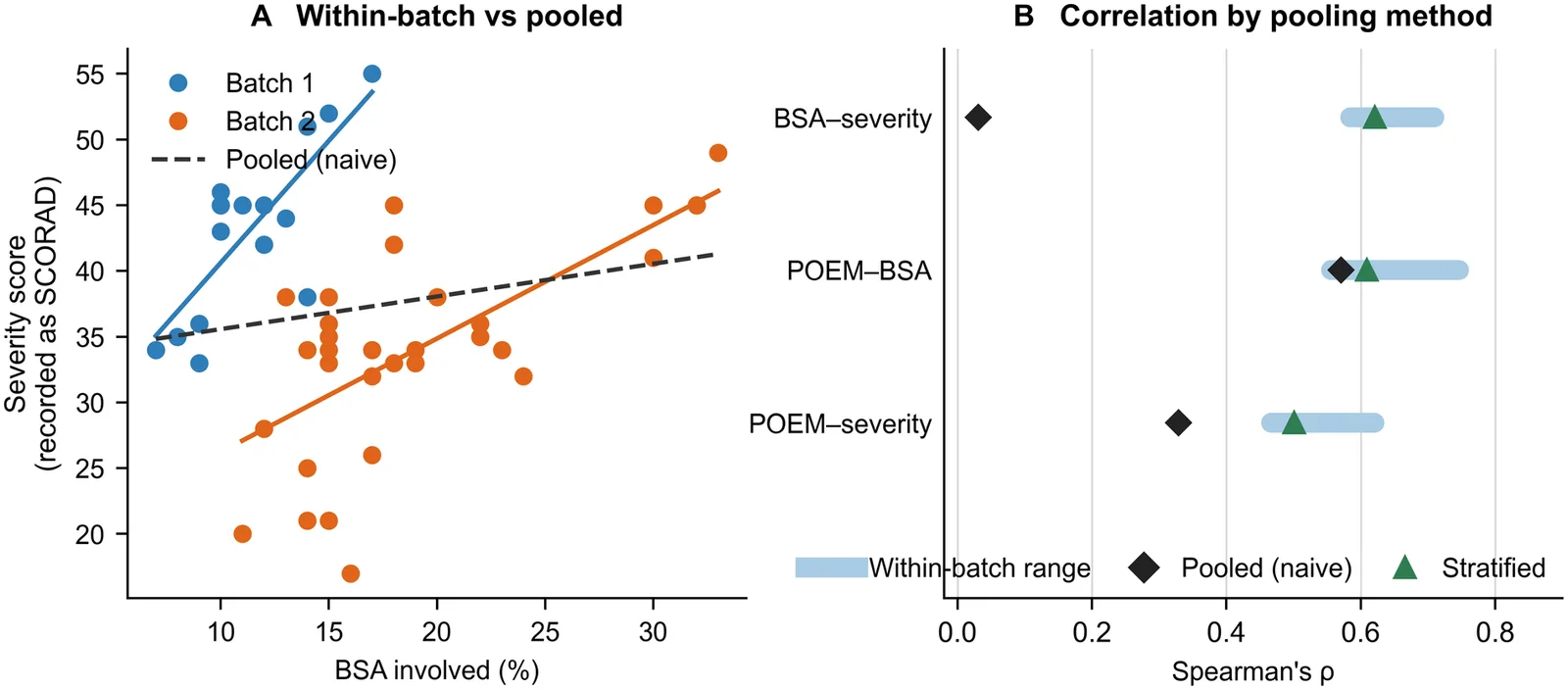
Body surface area, the clinician-assigned severity score and POEM in the two source-record batches (batch 1, *n* = 16; batch 2, *n* = 31). Both batches were assessed by the same dermatologist, who assigned the severity score as a global clinical impression rather than computing it from the SCORAD components (Section 2.2). **(A)** The severity score plotted against BSA: the two correlate steeply within each batch (regression lines) but the naive pooled regression (dashed) is almost flat, because batch 2 records had larger BSA yet a lower severity score, so the within-batch association is cancelled on aggregation. **(B)** Spearman correlations for three pairs of measures under different pooling. The within-batch range is shown as a band; the naive pooled estimate as a diamond; the stratified (within-batch rank) estimate as a triangle. The association between BSA and the severity score collapses to *ρ* = 0.03 on naive pooling and is 0.62 under stratification; the patient- or carer-reported POEM–BSA correlation does not collapse (0.57 pooled, 0.61 stratified), and POEM–severity collapses only partially (0.33 pooled, 0.50 stratified). Clinician-assessed severity is reported by batch throughout (Section 3.3).

Two further observations bear on this. First, POEM, which is patient- or carer-reported and therefore not subject to clinician rater drift, correlated with BSA within each batch (*ρ* = 0.75 and 0.56) and across the pooled cohort (*ρ* = 0.57, *p* < 0.001), whereas the POEM–severity correlation fell upon naive pooling (from *ρ* = 0.62 and 0.47 within-batch to 0.33); only the relationships involving the clinician-assigned score collapsed on pooling (Figure 2B). Second, the difference is not explained by the batches differing in age: batch 1 was older (median 13.0 vs. 10.0 years, *p* = 0.026), but after ranking within the three age groups used above, batch 2 retained both the higher BSA (*p* < 0.001) and the lower severity score (*p* = 0.005). Because the same dermatologist assessed both record sets, the between-batch difference cannot be attributed to a change of assessor or of instrument. These observations are the reason we report clinician-assessed severity separately by batch; they do not establish why one assessor's global scoring differed between the two sets, and we present no findings about SCORAD as an instrument.

We therefore report clinician-assessed severity by batch (Table 2). A stratified estimate that ranks BSA and the severity score within-batch before pooling gives *ρ* = 0.62, and an inverse-variance-weighted combination of the two within-batch coefficients gives *ρ* = 0.63 (95% CI 0.41–0.78); we report this in preference to the naive pooled value, while noting that it estimates only the rank association common to the two batches and removes by construction the twofold difference in their regression slopes (1.85 and 0.86 points per BSA percentage point).

In batch 1, the median severity score was 44.5 (IQR 37.5–45.2; range 33–55) and median BSA was 10.5% (IQR 9.8–13.2; range 7–17), with 13 moderate and 3 severe cases and no mild cases. In batch 2, the median severity score was 34.0 (IQR 32.0–38.0; range 17–49) and median BSA 17.0% (IQR 15.0–22.0; range 11–33), with 4 cases in the mild band, 27 moderate, and none severe. Median POEM was 15.0 in batch 1 and 16.0 in batch 2 (*p* = 0.20). Severity did not differ detectably across the three age groups (Kruskal–Wallis severity score *p* = 0.45, BSA *p* = 0.67); with 11, 17, and 19 patients per group, this does not exclude a clinically relevant difference.

Allergic rhinitis was not associated with the severity score in the pooled data (38.0 vs. 34.5, *p* = 0.076), nor within either batch (batch 1 *p* = 0.502; batch 2 *p* = 0.094).

The quality-of-life totals ranged from 11 to 37 after the provenance corrections of Section 3.1, on an instrument whose maximum is 30, and none fell below 11 (12–37 as originally entered in the source workbook). Prompted by that pattern, the assessing dermatologist retrieved the paper questionnaire and scoring template used at the time. The assessing clinician reported that both documents showed that the instrument administered was the Children's Dermatology Life Quality Index (8), abbreviated to “DLQI” in the summary workbook, and that its 10 items had been scored 1–4 rather than the standard 0–3, giving a recorded range of 10–40. We have not seen these documents; this is a retrospective account by the same clinician who assigned the severity score (Section 2.2), and the source records do not corroborate it. According to that account, the two values above 30 are correctly summed totals rather than transcription errors. Subtracting 10 from every record converts the totals to the standard 0–30 scale, and we report them on that scale throughout: median 8 (IQR 6–11, range 1–27, *n* = 47). Eight of the 47 totals were not taken from the summary workbook but retrieved from the original electronic outpatient record during the provenance check (Section 3.1); the assessing clinician confirmed that the electronic record stored the same unconverted 1–4 item totals as the workbook, so one conversion applies to all 47. Unlike the clinician-assigned severity score, the CDLQI did not differ between the two batches (median 8 in each, *p* = 0.28; Table 2), and it is summarised for the cohort as a whole in Table 1. All 47 patients were evaluable; 1 score fell in the “no effect” band, 15 in “small”, 23 in “moderate”, 6 in “very large,” and 2 in “extremely large.” The cohort includes three children under 4 years, for whom the Infants’ Dermatitis Quality of Life Index would ordinarily be preferred, and seven aged 16 years or older; the same paediatric instrument was used throughout, which limits comparability at both ends of the age range. CDLQI values are therefore reported on the standard scale and are interpretable, with the age range caveat above; POEM, scored on the same visits, is reported alongside them.

## 4 Discussion

### 4.1 Principal findings

The observations summarised here are descriptive, come from a single centre, and should be read as preliminary. In 47 children and adolescents with AD attending a tertiary dermatology clinic in southeastern China, house dust mite was the most frequently reported trigger, a family history of allergic disease was recorded in almost all patients (95.7%), and 7 in 10 had a recorded history of allergic rhinitis while recorded asthma was almost absent, which we interpret as a feature of the records rather than of the population. Disease was predominantly moderate within each batch, based on the SCORAD bands used here only as descriptive cut-offs for a globally assigned score (Section 2.2).

### 4.2 Triggers and the regional context

House dust mite predominance is consistent with a large Chinese paediatric AD series, a retrospective study of 8,797 children in Hangzhou in which house dust mite was the dominant sensitiser at 45.6% and carried both the highest median specific IgE and the highest proportion of high-level sensitisation (4). Our figure of 57.4% is higher, but the two quantities are not directly comparable: theirs is laboratory-confirmed specific IgE, whereas ours is clinician-recorded history. Reported triggers systematically over-represent foods and under-represent aeroallergens relative to testing, so the concordance in identifying house dust mite as the leading trigger is the meaningful observation rather than the difference in magnitude. Fujian's humid subtropical climate is favourable to mite proliferation, and a damp environment was itself reported as a trigger by more than a fifth of this cohort.

Food triggers—seafood or crustaceans in 40.4%, and egg or milk in 14.9%—were reported frequently. These are reported associations only: none was confirmed by specific IgE, skin prick testing, or oral challenge, and they are not evidence of food allergy or of sensitisation. We emphasise that reported food triggers in AD greatly exceed the prevalence of challenge-confirmed food allergy, and these data must not be used to support dietary elimination. Parental perception of food-triggered eczema is common, and elimination diets are widely self-initiated (9); yet unnecessary exclusion carries real nutritional risk in growing children and requires dietetic supervision when genuinely indicated (10). Nothing in these data identifies a food as causal in any individual patient.

### 4.3 Comorbidity pattern and the asthma figure

A recorded history of allergic rhinitis was present in seven of 10 patients in this cohort, and patients with such a history were older, a gradient compatible with the atopic march. This is a count of what was written in the outpatient records, not a verified comorbidity frequency, and the comparisons with cohort studies below are made for orientation only. In a cohort of 27,228 children with early-onset AD compared with 55,174 controls, allergic rhinitis developed in 28.3% vs. 12.6%, and severe AD was itself a risk factor for progression to the full atopic triad (11). The classical sequential march does not fit all patients; for instance, among 177,081 children newly diagnosed with AD, the onset pattern of atopic comorbidities varied substantially with age at diagnosis (12).

The single recorded case of asthma is a measure of ascertainment, not of prevalence. Its 95% confidence interval is 0.4%–11.8%. We make no comparison with published comorbid asthma frequencies, because this figure counts entries in dermatology records and is not a prevalence estimate. This was a dermatology rather than an allergy service; comorbidities were extracted from routine records not designed for research; no spirometry or bronchial challenge was performed; and respiratory or paediatric records were not cross-checked. We draw no clinical inferences from the figure, and we retain it precisely because it quantifies the limitation. The same argument applies to allergic conjunctivitis, recorded in 0 of 16 patients in batch 1 and 6 of 28 in batch 2.

The 95.7% family history of allergic disease is high relative to published cohorts, and the two high proportions in this cohort arose in different ways. The UK Working Party criteria used to enrol this cohort require an itchy skin condition plus three or more of five further items, one of which is a personal history of asthma or hay fever; a family history of atopic disease substitutes for that item only in children aged under 4 years, of whom there were three here. A recorded personal history of rhinitis is therefore partly constitutive of the diagnosis (incorporation bias), which inflates the 70.2% proportion. The family history figure is not: for 44 of the 47 patients, a family history could not contribute to the diagnosis at all. It is inflated instead by our deliberately broad definition—any allergic condition, including urticaria, in a relative of any degree, unverified, with four positives resting on urticaria alone—and by recall bias in parents attending specifically because of an atopic complaint. Neither figure is comparable with cohorts using an atopy-restricted definition. Selection is a further reason for caution: a tertiary dermatology clinic will over-represent families in which allergic disease is already recognised.

### 4.4 Severity scoring, and what we could not analyse

The relationship between BSA and the severity score differed between the two source-record batches in a way that is difficult to reconcile with a stable scoring practice. The assessing dermatologist reported that she scored both record sets herself and that the severity score was assigned as a global clinical impression rather than computed from the SCORAD components (Section 2.2). On that account, neither a change of assessor nor a change of instrument accounts for the difference, although the account rests on recollection and is not documented. What remains is a change over time in how one assessor applied a global scale, or a difference in case mix that we could not measure; we present neither as established. Two observations bear on it (Section 3.3): patient- or carer-reported POEM retained its association with BSA across the pooled cohort while the relationships involving the clinician-assigned score did not, and the difference survived ranking within age groups, so it is not a consequence of the batches differing in age. Intra-rater reliability of AD severity instruments is imperfect even among trained assessors (13)—the relevant consideration here, since one assessor scored both sets—although that study assessed objective SCORAD in adults and therefore provides no information regarding the pruritus and sleep-loss items, which objective SCORAD excludes by definition. We cannot exclude a genuine difference in case mix beyond age, which we were able to test and which did not account for the difference.

We therefore report clinician-assessed severity by batch and give a stratified estimate rather than claiming that no combined estimate is possible. This limits direct comparability with other series and is a genuine weakness of the dataset. We do not present the between-batch difference as a finding about SCORAD as an instrument, and because the recorded scores were not computed from the SCORAD formula, we do not treat them as interchangeable with SCORAD totals reported elsewhere.

### 4.5 Strengths and limitations

The main strengths are that every reported figure is derived from a single, internally consistent dataset with denominators stated for each variable; that severity is reported in the form the data can support rather than in the more comparable pooled form; and that measured but uninterpretable data are declared rather than omitted.

The limitations are substantial. The corrections described in Section 3.1 were verified against the original electronic outpatient records by the assessing clinician and were not independently audited; no second coder repeated the transcription, and the provenance check therefore establishes the internal consistency of the two source workbooks rather than the accuracy of the records themselves. This is a single-centre, cross-sectional study of 47 patients, without a control group, and the findings describe a tertiary dermatology outpatient population rather than the community. Every proportion reported here carries a wide confidence interval; the study is not powered to detect associations of moderate strength (Section 2.3), and all results should be treated as preliminary observations describing one clinic population rather than as generalisable estimates. A recorded history of rhinitis is partly constitutive of the Williams diagnostic criteria used for enrolment, so that proportion cannot be treated as an independent observation; the family history proportion is inflated instead by our broad definition and by recall bias (Section 4.3). The cohort contains no infants and no child aged under 3.6 years, and therefore does not cover the age range in which AD prevalence peaks. Triggers, comorbidities, and family history were all extracted from routine records without prospective operational definitions, and were not confirmed by specific IgE, skin prick testing, or standardised diagnostic assessment. Asthma and conjunctivitis were almost certainly under-ascertained, and their recording differed between the two batches; their status was missing for the same three patients, consecutive in the record sequence, indicating a block of incomplete records rather than random missingness. The two batches were assembled 46 days apart based on the only dating evidence available. Both were assessed by the same dermatologist, who reported having assigned the severity score as a global clinical impression rather than computing it from the SCORAD components—an account given retrospectively that the source records do not document; furthermore, the assessment was unblinded, no patient was scored by a second assessor, and no reliability data exist, so the contribution of scoring variability to the observed between-batch difference cannot be quantified and no combined severity statistic is offered as a primary estimate. The quality-of-life totals were recorded on a non-standard 1–4 item scale, which the assessing clinician reported having confirmed against the original questionnaire and scoring template, and were converted here to the standard 0–30 scale (Section 3.3). We have not seen either document, so the absolute CDLQI values and the band distribution should be read as conditional on that account; the conversion applied to all 47 totals preserves every rank. A serum IgE result is present in the routine records but is labelled only as “allergen IgE”; the assay platform, the analytical method, and the reference interval could be established neither from the source documents nor on enquiry within the hospital, and the two source batches carry different recorded reference limits. We therefore do not report these results, and this study contributes no laboratory data. The exclusion of patients on systemic therapy removed the most severe end of the spectrum. Eosinophil data were too incomplete to analyse. The age range, 3.6–19.0 years, is wide, and the cohort includes nine patients aged 15 years or older, so the label “children and adolescents” spans developmental stages with different AD phenotypes (3).

### 4.6 Conclusion

In this single tertiary dermatology clinic in southern China, the triggers most often documented were house dust mite, seafood or crustaceans, and a damp environment; a recorded history of allergic rhinitis was common, in part because a personal history of asthma or hay fever is one of the enrolment criteria, and a family history of allergic disease was recorded in almost all patients under a deliberately broad definition; and clinician-assessed disease was predominantly moderate within each batch, based on bands used only as descriptive cut-offs (Section 2.2). The very low recorded asthma frequency is a record-keeping artefact in a dermatology setting and not a population characteristic; we do not use it to quantify the extent of under-recording. All of these are recorded-history observations from 47 patients at a single centre. They are preliminary; they are not prevalence estimates or confirmed sensitisation frequencies, and they require confirmation in larger, prospectively defined, multi-centre cohorts with confirmed sensitisation testing, a documented severity scoring procedure, and a specified laboratory panel.

## Data Availability

The analysis code that generates the results reported in this article, with the exception documented in Section 2.3, is provided as Supplementary Material, together with the trigger coding dictionary (Supplementary Table 2) and a variable-by-variable data dictionary (Supplementary Table 3). The underlying dataset contains individual-level clinical data from minors; the ethics approval for this study (approval no. (伦审)2026(科研)第060号 (English translation: Ethics Review 2026, Research, No. 060)) covers a retrospective analysis of de-identified, routinely collected records and does not extend to public deposition of the individual-level data. The same reasoning applies to the two source-position matrices used by the provenance check of Section 3.1, which were therefore supplied to the Editorial Office for the reviewers rather than published. A de-identified dataset that reproduces the reported analyses, subject to that exception, is available to qualified researchers on submission of a research proposal to the corresponding author and approval by the same Ethics Committee; requests will be answered within 30 days.
