# Supplementary material for: Clinical profile, reported triggers, and comorbidity pattern in children and adolescents with atopic dermatitis: a cross-sectional study from Fujian, China
Source: Front Pediatr. 2026 Sep 23;14:1946416. doi: 10.3389/fped.2026.1946416 (PMC13642631; doi:10.3389/fped.2026.1946416)
Supplement: Supplementary file 1 [file Table1.docx]

# Supplementary Table 1. STROBE Statement — checklist of items that should be included in reports of cross-sectional studies

| No. | Item | Recommendation | Reported in |
| --- | --- | --- | --- |
| 1 | Title and abstract | (a) Indicate the study design in the title or abstract | Title; Abstract, Methods |
|  |  | (b) Provide in the abstract an informative and balanced summary | Abstract |
| 2 | Background/rationale | Explain the scientific background and rationale | Section 1, paragraphs 1–3 |
| 3 | Objectives | State specific objectives | Section 1, final paragraph |
| 4 | Study design | Present key elements of study design early in the paper | Section 2.1, first sentence; Abstract, Methods |
| 5 | Setting | Describe setting, locations, and relevant dates including periods of recruitment and data collection | Section 2.1, including the dates and composition of the two source-record batches |
| 6 | Participants | Give the eligibility criteria, and the sources and methods of selection of participants | Section 2.1, inclusion and exclusion criteria |
| 7 | Variables | Clearly define all outcomes, exposures, predictors, potential confounders and effect modifiers; give diagnostic criteria | Section 2.2, including the trigger coding frame and the definitions of family history and of each comorbidity |
| 8 | Data sources/measurement | For each variable of interest, give sources of data and details of methods of assessment | Section 2.2 |
| 9 | Bias | Describe any efforts to address potential sources of bias | Section 2.1 (exclusion criteria and their effect on the severity spectrum); Section 2.3 (within-batch reporting); Section 4.3 (incorporation bias from the Williams criteria; ascertainment of comorbidities); Sections 3.3 and 4.4 (batch-related scoring difference); Section 4.5 |
| 10 | Study size | Explain how the study size was arrived at | Section 2.1 and Section 3.1 — the 49 records identified in the two source batches; no screening log exists; no a priori sample size calculation, as the study is descriptive |
| 11 | Quantitative variables | Explain how quantitative variables were handled in the analyses | Section 2.3; age groupings in Section 3.2 |
| 12 | Statistical methods | (a) Describe all statistical methods, including those used to control for confounding | Section 2.3 |
|  |  | (b) Describe any methods used to examine subgroups and interactions | Section 2.3 (within-batch analyses); Section 3.3 |
|  |  | (c) Explain how missing data were addressed | Section 2.3 (denominators stated per variable); Table 1 |
|  |  | (d) If applicable, describe analytical methods taking account of sampling strategy | Not applicable |
|  |  | (e) Describe any sensitivity analyses | Section 3.1 (provenance check; the sensitivity analysis setting the residual same-position values to missing; and, for the six trigger entries flagged by the comparison and subsequently verified, the adversarial bound reported alongside them); Section 3.3 (within- vs between-batch) |
| 13 | Participants | (a) Report numbers of individuals at each stage of the study | Section 3.1 |
|  |  | (b) Give reasons for non-participation at each stage | Section 3.1 |
|  |  | (c) Consider use of a flow diagram | Not used; the series comprises 49 identified patients, 2 excluded, 47 analysed (Section 3.1) |
| 14 | Descriptive data | (a) Give characteristics of study participants and information on exposures and potential confounders | Table 1; Section 3.2 |
|  |  | (b) Indicate number of participants with missing data for each variable of interest | Table 1 (“n with data” column) |
| 15 | Outcome data | Report numbers of outcome events or summary measures | Tables 1 and 2; Sections 3.1–3.3 |
| 16 | Main results | (a) Give unadjusted estimates and, if applicable, confounder-adjusted estimates and their precision | Tables 1 and 2, with Wilson 95% CIs for key proportions |
|  |  | (b) Report category boundaries when continuous variables were categorized | Section 3.2 (age groups); Section 2.2 (SCORAD severity bands) |
|  |  | (c) If relevant, consider translating estimates of relative risk into absolute risk | Not applicable |
| 17 | Other analyses | Report other analyses done | Section 3.3 (association not reproducible within batch, reported as such and not claimed) |
| 18 | Key results | Summarise key results with reference to study objectives | Section 4.1 |
| 19 | Limitations | Discuss limitations, taking into account sources of potential bias or imprecision | Sections 4.3, 4.4 and 4.5 |
| 20 | Interpretation | Give a cautious overall interpretation of results | Sections 4.2–4.4; Section 4.5 |
| 21 | Generalisability | Discuss the generalisability of the study results | Sections 4.5 and 4.6 |
| 22 | Funding | Give the source of funding and the role of the funders | Funding declaration |

An Explanation and Elaboration article discusses each checklist item and gives methodological background and published examples of transparent reporting. The STROBE checklist is best used in conjunction with this article (freely available at https://www.strobe-statement.org).
