# Supplementary material for: Clinical profile, reported triggers, and comorbidity pattern in children and adolescents with atopic dermatitis: a cross-sectional study from Fujian, China
Source: Front Pediatr. 2026 Sep 23;14:1946416. doi: 10.3389/fped.2026.1946416 (PMC13642631; doi:10.3389/fped.2026.1946416)
Supplement: Supplementary file 2 [file Table2.docx]

**Supplementary Table 2.** Dictionary used to code free-text entries in the routine “allergen” field into the eleven trigger categories. The field was completed for all 47 patients, and 47 is the denominator for every proportion reported. Coding was performed by a single investigator; the dictionary was not registered in advance and is published here so that each decision can be inspected. Counts are on the corrected data (Section 3.1). Six second-batch entries carry a same-position agreement with the first batch that the comparison of Section 3.1 flagged: three are identical token for token to the entry at the same row position, and two coded categories in this table (egg or milk, animal dander) show a same-position excess. All six were put to the assessing clinician and confirmed against the original electronic outpatient records; no count in this table was changed as a result.

| **Category (as reported)** | **Source term(s)** | **English gloss** | **Patients, n (of 47)** |
| --- | --- | --- | --- |
| House dust mite | 尘螨 | dust mite | 27 |
| Seafood or crustaceans | 海鲜 / 虾蟹 / 螃蟹 | seafood / shrimp and crab / crab | 19 |
| Damp environment | 潮湿 / 潮湿环境 | damp / damp environment | 10 |
| Egg or milk | 鸡蛋 / 牛奶 / 鸡蛋空气 | hen's egg / cow's milk / a garbled entry, see note | 7 |
| Pollen | 花粉 | pollen | 7 |
| Cold air | 冷空气 | cold air | 7 |
| Animal dander | 猫毛 / 狗毛 | cat hair / dog hair | 6 |
| Heat | 热 / 热刺激 | heat / heat stimulus | 6 |
| Beef or mutton | 牛肉 / 羊肉 / 牛羊肉 | beef / mutton / beef or mutton | 4 |
| Wheat | 小麦 | wheat | 1 |
| Cockroach | 蟑螂 | cockroach | 1 |

**Patients with no entry in any category (retained in the denominator as true zeros)**

| **Source term** | **English gloss** | **Patients, n** | **Note** |
| --- | --- | --- | --- |
| 未诉明确过敏原 | no definite allergen reported | 1 | An explicit negative entry in the original record. The workbook had carried a six-item entry copied from the first batch at the same row position; the provenance check identified it and it was corrected against the original record (Section 3.1). |
| 芒果 | mango | 1 | The only entry recorded for this patient. No fruit category had been defined. |

**Further entries not mapping to any category (five entries in four patients; those patients also report coded triggers)**

| **Source term** | **English gloss** | **Patients, n** | **Reason not coded, and effect** |
| --- | --- | --- | --- |
| 干燥 | dry environment | 2 | No category for a dry environment had been defined. |
| 毛发 | unspecified hair | 1 | Not coded as animal dander: the dictionary required a named animal (cat or dog). |
| 食物 | unspecified “food” | 1 | Assigned to none of the four food categories. |
| 空气 (residue of 鸡蛋空气) | “air”, the residue of a garbled entry | 1 | One record reads 鸡蛋空气 with no separator. The 鸡蛋 (egg) component was coded and the residue left uncoded; had it been intended as 冷空气 (cold air), Cold air would be 8 of 47 rather than 7. This is the one place where coding went beyond the term as written. |

Two patients therefore contribute a zero to all eleven categories, and five further entries in four patients were left uncoded. The eleven categories are exhaustive of what could be coded but not of what was reported: two uncoded entries (mango and an unspecified “food”) are food reports appearing in none of the four food categories, so category proportions are unbiased for the categories as defined but are lower bounds for broader groupings such as “any food trigger”. Three categories — damp environment, heat and cold air — are non-specific aggravating factors rather than candidate allergens. All entries are clinician-documented, parent- or patient-reported associations; none was confirmed by specific IgE testing, skin prick testing or oral challenge.
