# Supplementary material for: Clinical profile, reported triggers, and comorbidity pattern in children and adolescents with atopic dermatitis: a cross-sectional study from Fujian, China
Source: Front Pediatr. 2026 Sep 23;14:1946416. doi: 10.3389/fped.2026.1946416 (PMC13642631; doi:10.3389/fped.2026.1946416)
Supplement: Supplementary file 3 [file Table3.docx]

**Supplementary Table 3.** Data dictionary for the de-identified analysis dataset (47 rows, one per patient) consumed by the analysis code (Supplementary Data Sheet 1). All values are those after the corrections described in Section 3.1.

| **Variable** | **Values** | **Definition and provenance** | **n with data** |
| --- | --- | --- | --- |
| id | string | De-identified patient identifier (P001–P047). | 47 |
| batch | 1 / 2 | Source-record batch. The records carry no dates; the batches are defined by the two source files. | 47 |
| age_band | <6 / 6–11 / 12–15 / 16–19 | Age at index visit, banded for de-identification. Exact age is not released. | 47 |
| sex | M / F | Recorded sex. Missing in one record whose field held an uninterpretable entry. | 46 |
| residence | urban / rural | Recorded place of residence. | 47 |
| rhinitis | 0 / 1 | Affirmative entry for allergic rhinitis in the routine clinical history. Recorded history, not a verified diagnosis. | 47 |
| asthma | 0 / 1 | Affirmative entry for asthma. Missing for three consecutive records in batch 2 whose fields are illegible. | 44 |
| conjunctivitis | 0 / 1 | Affirmative entry for allergic conjunctivitis. Same three records missing. | 44 |
| family_atopy | 0 / 1 | Any allergic condition, including urticaria, in a relative of any degree; unverified. | 47 |
| trig_* (11 columns) | 0 / 1 | One column per trigger category (Supplementary Table 2). The field was completed for every patient; two patients score zero in all eleven. | 47 |
| BSA_pct | numeric | Body surface area involved at the index visit, per cent. | 47 |
| severity_score | numeric | Clinician-assigned severity score, recorded in the source workbook under the SCORAD label. The assessing dermatologist reports having assigned it as a global clinical impression rather than computing it from the SCORAD components (Section 2.2). | 47 |
| POEM | numeric | Patient- or carer-reported Patient-Oriented Eczema Measure. | 46 |
| CDLQI | numeric | Children's Dermatology Life Quality Index, converted to the standard 0–30 scale by subtracting 10 from the recorded total (the assessing clinician reports that its ten items were scored 1–4 and confirms that the electronic record, the source of eight of the 47 totals, stores the same unconverted totals; Section 3.3). | 47 |
| weight_kg | numeric | Body weight at the index visit, kilograms. | 47 |

Exact age is released only in bands, so the age descriptive statistics in Table 1 and every comparison using exact age cannot be reproduced from this dataset; the rhinitis–age comparisons are reproduced on bands instead (pooled p = 0.020 against 0.013 on exact ages; within batch p = 0.251 and p = 0.059 against p = 0.200 and p = 0.042). Every other result reported from this dataset is produced by the accompanying code, from this dataset or, for the provenance check of Section 3.1, from the two source-position matrices supplied with this article.
